# An orange fluorescent protein tagging system for real-time pollen tracking

**DOI:** 10.1186/1756-0500-6-383

**Published:** 2013-09-27

**Authors:** J Hollis Rice, Reginald J Millwood, Richard E Mundell, Orlando D Chambers, Laura L Abercrombie, H Maelor Davies, C Neal Stewart

**Affiliations:** 1 Department of Plant Sciences, University of Tennessee, 37996, Knoxville, TN, USA; 2 Kentucky Tobacco Research & Development Center, University of Kentucky, 40546 Lexington, KY, USA; 3 Department of Plant & Soil Sciences, University of Kentucky, 40546 Lexington, KY, USA

**Keywords:** Gene flow, Male-sterility, Plant made pharmaceuticals, Bioconfinement, Nicotiana, Green fluorescent protein (GFP), Orange fluorescent protein (OFP)

## Abstract

**Background:**

Monitoring gene flow could be important for future transgenic crops, such as those producing plant-made-pharmaceuticals (PMPs) in open field production. A *Nicotiana* hybrid (*Nicotiana. tabacum × Nicotiana glauca)* shows limited male fertility and could be used as a bioconfined PMP platform. Effective assessment of gene flow from these plants is augmented with methods that utilize fluorescent proteins for transgenic pollen identification.

**Results:**

We report the generation of a pollen tagging system utilizing an orange fluorescent protein to monitor pollen flow and as a visual assessment of transgene zygosity of the parent plant. This system was created to generate a tagged *Nicotiana* hybrid that could be used for the incidence of gene flow. *Nicotiana tabacum* ‘TN 90’ and *Nicotiana glauca* were successfully transformed via *Agrobacterium tumefaciens* to express the orange fluorescent protein gene, *tdTomato-ER*, in pollen and a green fluorescent protein gene, *mgfp5-er*, was expressed in vegetative structures of the plant. Hybrids were created that utilized the fluorescent proteins as a research tool for monitoring pollen movement and gene flow. Manual greenhouse crosses were used to assess hybrid sexual compatibility with *N. tabacum*, resulting in seed formation from hybrid pollination in 2% of crosses, which yielded non-viable seed. Pollen transfer to the hybrid formed seed in 19% of crosses and 10 out of 12 viable progeny showed GFP expression.

**Conclusion:**

The orange fluorescent protein is visible when expressed in the pollen of *N. glauca, N. tabacum,* and the *Nicotiana* hybrid, although hybrid pollen did not appear as bright as the parent lines. The hybrid plants, which show limited ability to outcross, could provide bioconfinement with the benefit of detectable pollen using this system. Fluorescent protein-tagging could be a valuable tool for breeding and *in vivo* ecological monitoring.

## Background

Increased use of transgenic crops has prompted the necessity of monitoring transgene flow in agroecological systems. Previous investigations have ascertained the utility of gene flow tracking with fluorescent proteins (FPs) [1-4]. These studies have shown that green fluorescent protein (GFP) is an effective tool for the purpose of gene flow tracking and can be targeted to various organs and tissues within plants, including pollen. This technology, in effect, could be used in an environmental monitoring system, one of the many uses of FPs in plants [5]. One drawback of using native GFP as a marker in plants is the signal-to-noise ratio at GFP’s maximum excitation wavelength of 395 nm, often resulting in autofluorescence of plant tissue components [6]. Fluorescent proteins emitting in the red/orange spectrum that require longer wavelengths for excitation have lower levels of autoflorescence in plant tissues compared to blue or UV light [6]. One such widely used orange fluorescent protein (OFP), DsRed, is derived from *Discosoma* sp. its mutant variants have higher extinction coefficients and quantum yields [7]. Coral-derived FPs should be useful for monitoring gene flow.

*Nicotiana tabacum* (tobacco) and *Brassica napus* (canola) plants have been transformed to synthesize GFP in pollen, using pollen-specific promoters [1,4]. Long-range pollen tracking was conducted in canola species to assay pollen movement in real time (e.g. immediate detection of tagged pollen) using traps at various distances within field and greenhouse experiments. This method is quicker and less laborious for determining pollen flow than analyzing progeny from recipient plants (e.g. antibiotic screening, PCR, FP screening) [8]. Drawing upon this previous body of work, it is logical to conceptualize a method to determine bioconfinement efficacy using FP tagging utilizing an improved fluorescent protein for plants.

The *Nicotiana* hybrid (*Nicotiana tabacum* × *Nicotiana glauca*), is highly sterile and prompted a further examination of bioconfinement through gene flow monitoring. Recently, we have shown that GFP tagging in vegetative plant tissues of this hybrid allows for gene tracking and assists with sterility assessments [9]. Here we describe a modified system to tag pollen that is applicable to a real-time assay of pollen flow from FP-tagged plants. Our goal was to engineer each *Nicotiana* species for pollen-specific expression of an OFP gene that also had vegetative tissues that expressed a GFP gene. The transgenic plants could then be crossed to obtain interspecific hybrid *Nicotiana*s that had FP genes contributed from each parent. To achieve this goal, parent plants *N. tabacum* ‘TN 90’ and *N. glauca* were *Agrobacterium*-mediated transformed to synthesize the OFP tdTomato-ER in the pollen and bred to homozygosity, and then crossed to create the transgenic interspecific hybrid. Manual greenhouse crosses were performed to assess sexual compatibility and functionality of the system.

## Methods

### Plants

*N. tabacum* ‘TN 90’ used for transformation was from foundation seed lot # 86-02-K-4A, *N. tabacum* ‘MS TN 90’ from foundation seed lot # 86-03-KLC-15 is a male sterile variety of TN 90 that was used as a pollen recipient plant in crosses. *N. tabacum* ‘SN 2108’’, a morphologically distinct variety from the TN 90 cultivar used as a pollen donor in greenhouse crosses, is an experimental line developed into ‘KT D4’; all *N. tabacum* were obtained from the Kentucky Tobacco Seed Improvement Association, Inc. in Lexington, KY, USA. (38°8’N, 84°29’W). *N. glauca* used for transformation was from the US National Plant Germplasm System (plant introduction 307908, accession TW55 from Peru).

### Transformation

#### Vector construction

**Figure 1:**
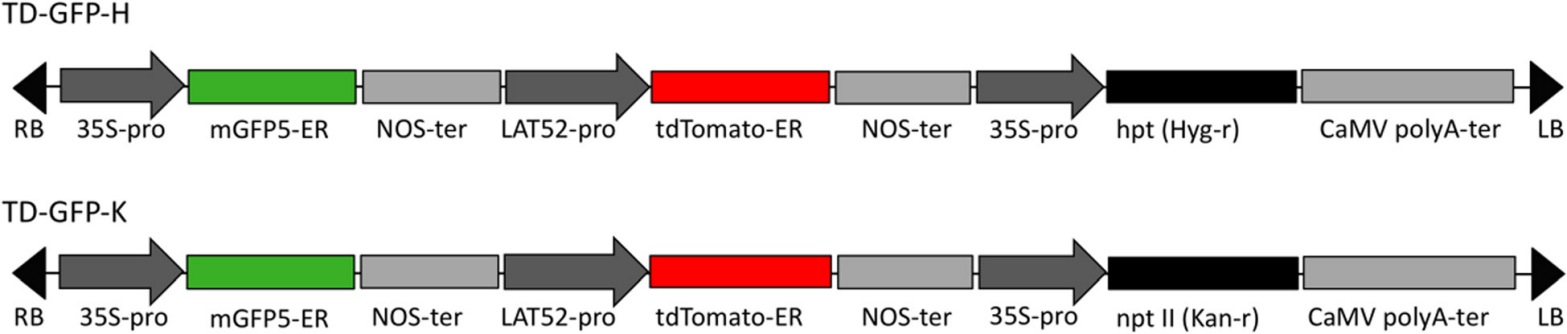
**Cassettes used in transformation.** Cassettes TD-GFP- H and TD-GFP-K were used in transformation of *N. glauca* and *N. tabacum* ‘TN 90’ with *A. tumefaciens* strain EHA105. Vectors are identical except for the *hpt* gene present in TD-GFP-H and the *npt II* gene present in TD-GFP-K.

Two fluorescent proteins were used to mark plants. The *mgfp5-er* gene encodes a GFP that emits green light (λ_max_ = 509 nm) when excited by wavelengths of blue (465 nm) or ultraviolet (UV; 395 nm) light and targeted and retained in the endoplasmic reticulum (ER) within cells. GFP in transgenic plants is observable by UV light illumination in the dark or epifluorescence microscopy, and is quantifiable using fluorescence spectrometry [10,11]. tdTomato-ER, is a *DsRed* variant that is a tandem dimer OFP (λ_max_ = 581 nm and excited by green light (554 nm) that is also retained in the ER [7,12]. To create dual FP marker vectors, the Gateway compatible vector backbone pMDC99, containing a hygromycin resistance cassette, and pMDC100, containing a kanamycin resistance cassette, were utilized as Gateway destination vectors [13]. An entry vector containing a pollen-specific promoter, *LAT52*[14], driving expression of the OFP *tdTomato-ER* and a nos terminator was recombined with the destination vectors, creating the intermediate vectors pMDC99-tdTomato-ER and pMDC100-tdTomato-ER. Subsequently a GFP expression cassette (containing CaMV35S-mGFP5-ER-nosT) was amplified from pBIN19-mGFP5-ER and cloned into the intermediate vectors, creating the binary vectors TD-GFP-H (containing hygromycin selection) and TD-GFP-K (containing kanamycin selection), respectively (Figure 1). These vectors were identical except for the antibiotic resistance genes to facilitate screening by using dual antibiotic selection after hybridization of *Nicotiana* species to incorporate both constructs into the F_1_ hybrid.

#### Generation of transgenic plants

**Figure 2:**
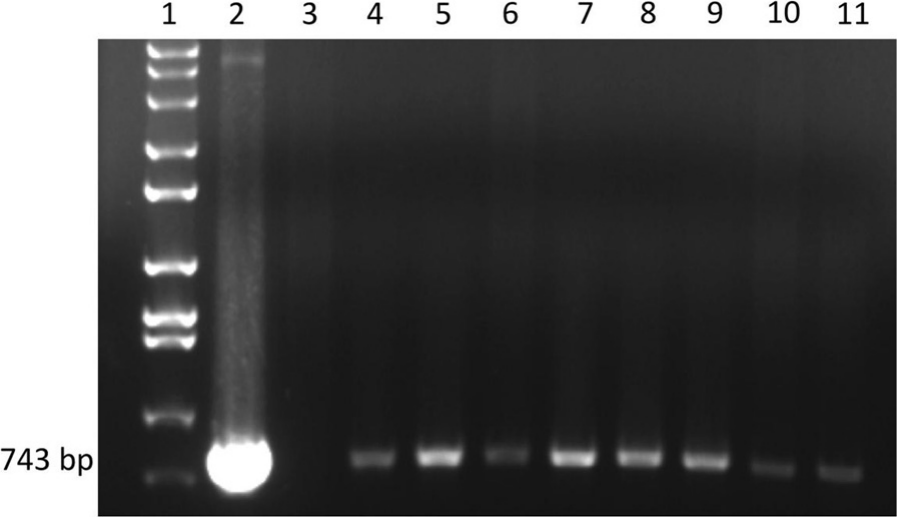
**PCR analysis of T_0_ TN 90 plants transformed with TD-GFP-H.** Lane 1: DNA marker, Lane 2: purified mGFP5ER plasmid as a positive control. Lanes 3–11: genomic DNA from putative transgenic TN 90. The 743 bp band present in lanes 4 through 11 confirmed transgene presence in plants. All other transgenic events showed similar results.

Plant transformation experiments were performed using *Agrobacterium tumefaciens* strain EHA 105 using the previously-described leaf disc method [15]. Sterilized leaf explants were soaked for 30 min in a mixture of *Agrobacterium* and liquid MS salts containing B_5_ vitamin (DBI). Transformed explants were then co-cultivated on solid DBI media for 2 days before being transferred to solid DBI containing Timentin® (400 mg/L) and either kanamycin (200 mg/L) or hygromycin (50 mg/L) for selection. Shoots generated from transformed callus were transferred to MS media containing respective selective antibiotics [15,16]. Shoots were maintained at 24°C under 16/8 h light/dark periods until rooting, then transferred to soil in standard 1020 flats divided in to 18 cells measuring 7.95 cm × 7.94 cm × 5.72 cm with humidity domes to allow for acclimation for approximately two weeks. Plants leaves were then assayed with a handheld UV light (UVP model B-100AP 100 W:365 nm) to detect differentiate between transgenic and non-transgenic plants as previously described [11]. The presence of the TD-GFP-H and TD-GFP-K inserts were confirmed in each T_0_ plant by DNA extraction and PCR to amplify *mgfp5-er* DNA (Figure 2) as previously described [1,17]. Plants that were confirmed visually and with PCR to be transgenic were transferred into 4 L pots in a greenhouse under 16/8 h light/dark periods at 27°/20°C, respectively. Seeds were harvested from each plant by covering flowers with breathable mesh pollination bags (DelStar Technologies, Inc., Middleton, DE, USA) and periodically manually shaken until seed pods developed and were harvested. In all, 20 events were generated per construct per species.

### Hybrid *Nicotiana* production

Plants were bred to obtain lines that had both constructs for a complete tracking of pollen. To ensure multiple transgene copies were stacked into the hybrid, our goal was to produce hybrids containing one TD-GFP-K and one TD-GFP-H construct, using dual antibiotic screening to ensure to select hybrids that were transgenic for each construct.

### Fluoresence measurements and observations

**Figure 3:**
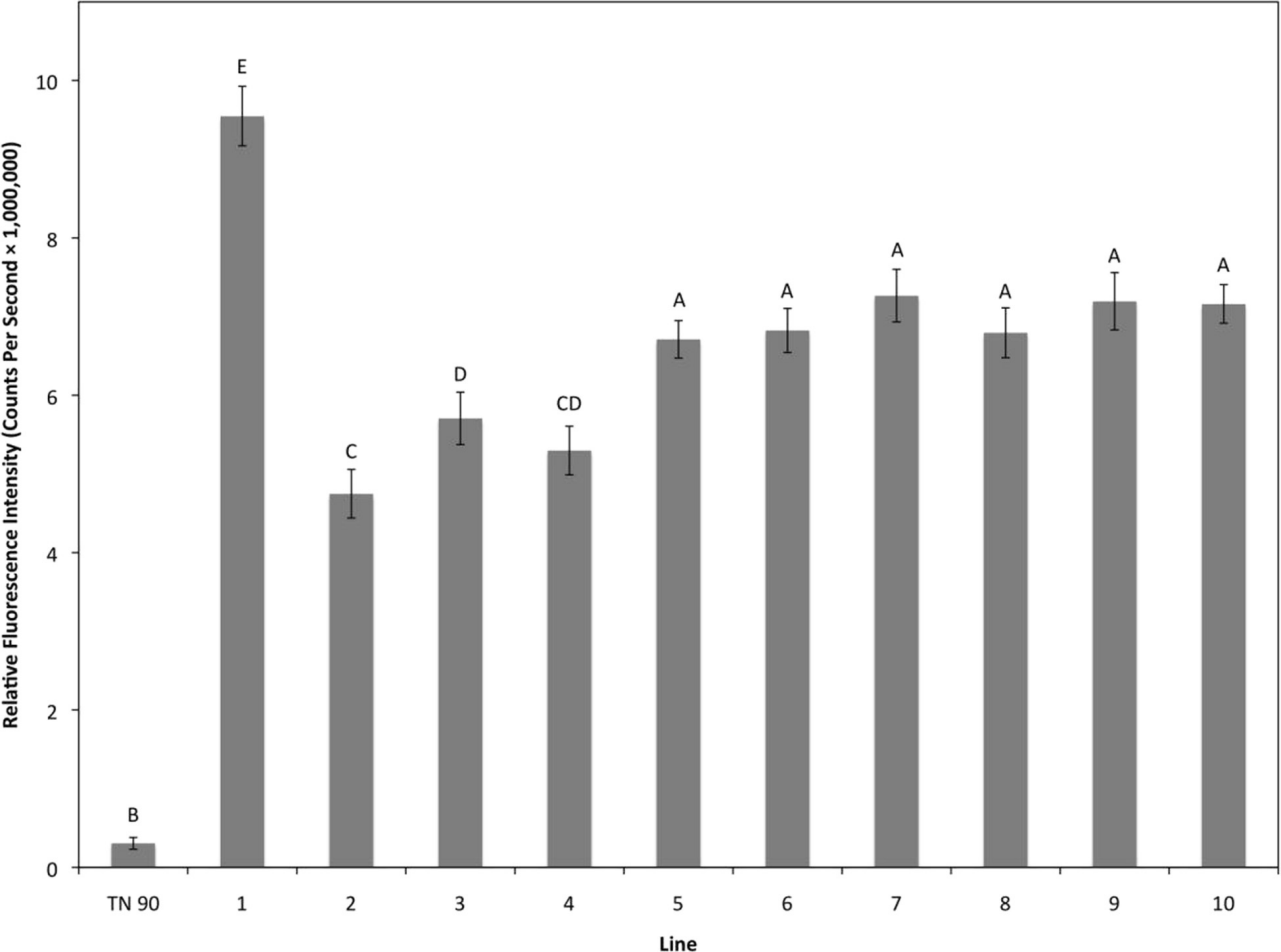
**Average relative fluorescence intensity for TN 90 TD-GFP-H T_1_ lines.** Leaf tissue from a non-transgenic TN 90 line and ten transgenic T_1_ lines were excited at 395 nm and measured at an emission of 509 nm with a spectrofluorometer. Fluoresence intensity values were normalized to an average measurement of TN 90 negative control plants outside the spectral range of GFP emission. For TN 90 n = 2, TD-GFP-H lines1-8, and 10 n = 34, line 9 n = 33. Error bars represent the standard error from the mean and different letters indicate significant differences at P < 0.05.

**Figure 4:**
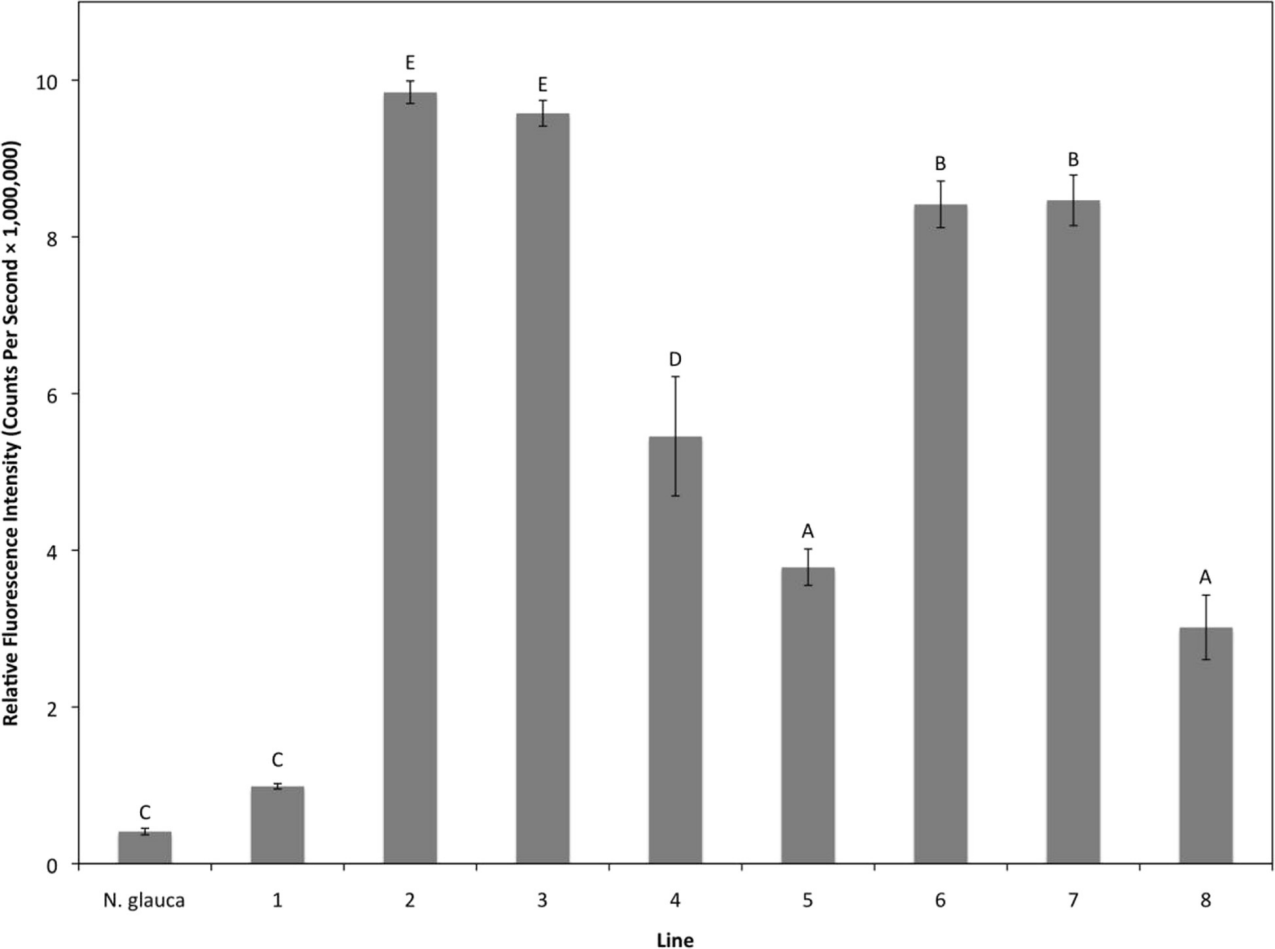
**Average relative fluorescence intensity for *N. glauca *TD-GFP-K T_1_ lines.** Leaf tissue from a non-transgenic *N. glauca* line and eight transgenic T_1_ lines were excited at 395 nm and measured at an emission of 509 nm with a spectrofluorometer. Fluoresence intensity values were normalized to an average measurement of *N. glauca* negative control plants outside the spectral range of GFP emission. For *N. glauca* n = 2, TD-GFP-K lines 1 n = 26, line 2 n = 34, line 3 n = 31, line 4 n = 16, lines 5 and 6 n = 33, line 7 n = 28, and line 8 n = 24. Error bars represent the standard error from the mean and different letters indicate significant differences at P < 0.05.

**Figure 5:**
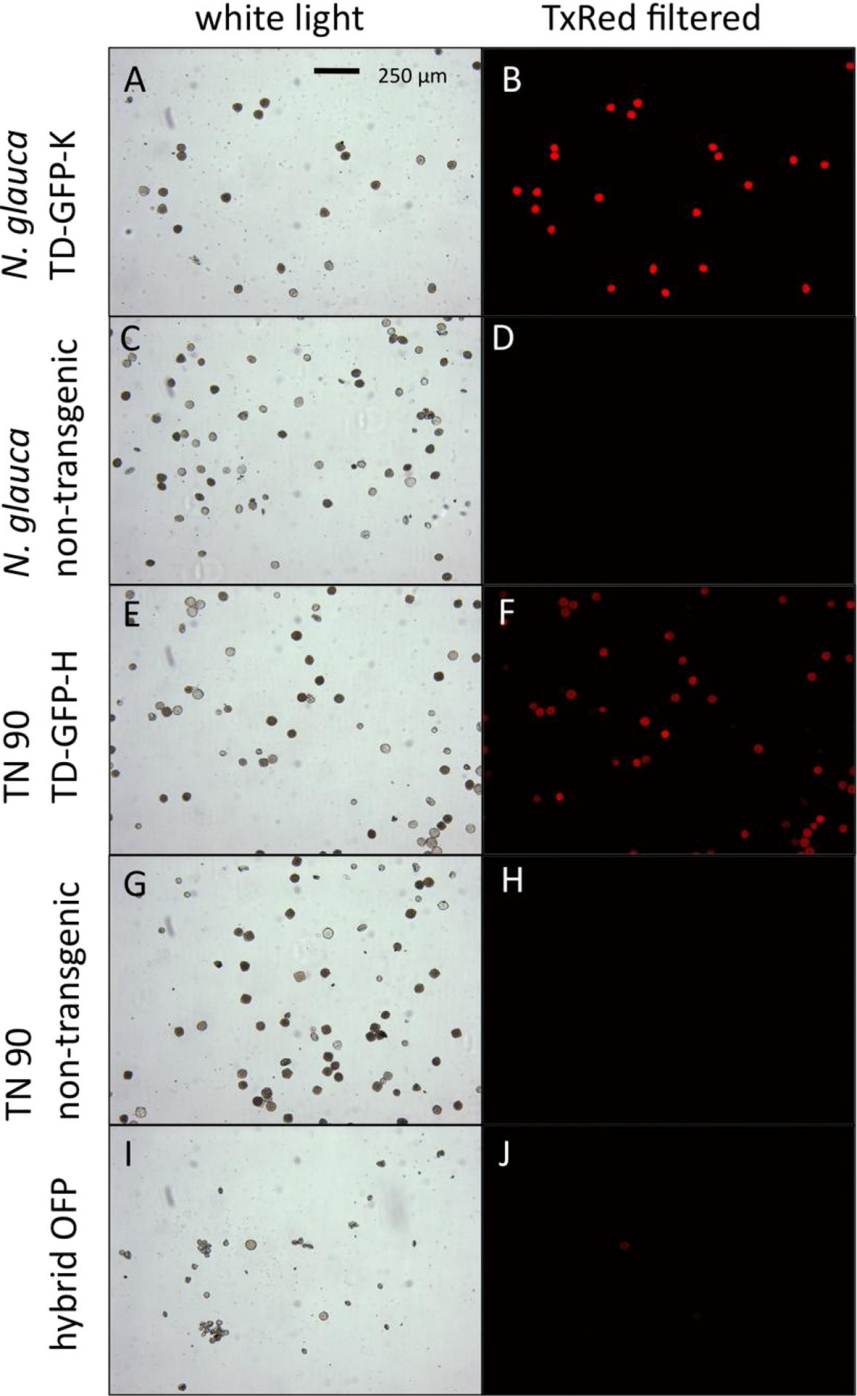
**Fluorescent protein visualization in pollen.** Orange fluorescent protein-tagged pollen as viewed under an epifluorescent microscope at 100×. *N. glauca* and TN 90 plants have been bred for homozygosity of tdTomato-ER, resulting in hybrid OFP plants. All white light images **(A)**, **(C)**, **(E)**, **(G)**, and **(I)** were captured at 80 ms exposure time. Panels **(B)**, **(D)**, **(F)**, **(H)**, and **(J)** are epifluorescent microscopy images using green light excitation and orange light emission. Panels **(B)** and **(D)** were captures at 50 ms exposure time. Panels **(F)** and **(H)** were captured at 80 ms exposure time. Panel **(J)** was captured at 180 ms exposure time.

Brightly fluorescent T_0_ plants, as determined by visual observation for GFP, were selected for analysis and further breeding; ten T_1_ TN 90 GFP-H lines and eight *N. glauca* T_1_ TD-GFP-K lines were selected. T_1_ seeds were germinated and handheld UV light was used to select the brightest GFP-expressing seedlings. GFP fluorescence was measured by a spectrofluorometer (Fluorolog®-3 HORIBA Jobin Yvon, Edison, NJ, USA) [11,18] and analyzed with its software (FluorEssence™ Version 2.5.2.0.HORIBA Jobin Yvon, Edison, NJ, USA) to quantify average fluorescence (photon counts per second) from each T_1_ line (Figures 3 and 4). Individual plants were selected that had the highest measured fluorescence, and thus, were most likely to be homozygous for the TD-GFP-K or TD-GFP-H inserts [18]. When T_1_ plants flowered, pollen was taken from each plant, suspended in 200 μl of water, and 15 μl of the suspension transferred to a microscope slide and observed under an epifluorescent BX 51 microscope (Olympus Corporation, Shinjuku, Tokyo, Japan). A Texas Red®/Cy3.5 (TxRed) filter set (Chroma Technology Corporation, Bellows Falls, VT, USA) was used to view fluorescent pollen grains. The field of view was captured by a digital camera (Olympus Q Color 3) and Qcapture imaging system (Q Imaging Corp., Burnaby, Canada) (Figure 5).

### Transgenic line selection

With the assumption of a single insertion event, transgene zygosity was estimated using epifluorescent microscopy. Plants with 100% fluorescent pollen (deemed homozygous) were bagged and self-fertilized as previously described. In addition to the FP pollen assay, we used progeny assays to assure that we selected homozygosity of each T_2_ line. Germinated seed were screened with a handheld UV light to determine zygosity of each T_2_ line (using ratios of GFP to non-GFP plants). Seeds of each T_2_ line were also screened for inheritance of antibiotic resistance genes by germination on MS media [15] containing kanamycin (200 mg/L) or hygromycin (50 mg/L) wherein observation of plant health allowed for determination of segregation; 100% survival indicated a homozygous line for selection. The selected transgenic T_2_ lines, TN 90 TD-GFP-H and *N. glauca* TD-GFP-K*,* were crossed (TN 90 TD-GFP-H × *N. glauca* TD-GFP-K). Hybrid lines derived from parents lines transformed with the TD-GFP-K/TD-GFP-H constructs were named ‘Hybrid OFP’ plants. These hybrid seeds were germinated on MSO media containing both kanamycin (200 mg/L) and hygromycin (50 mg/L) to ensure both constructs were integrated into the hybrid genome.

### Fertility assessment in hybrids

Manual crosses were conducted in a greenhouse in Lexington, Kentucky (Table 1). Hybrid OFP plants were crossed with the male sterile *N. tabacum* ‘MS TN 90’ to determine hybrid outcrossing potential and transgene transmission rates. To evaluate female fertility, SN 2108, a pollen donor was crossed to hybrid OFP plants, which were emasculated prior to crossing. For both types of crosses, 8 pairs of plants were crossed, with 12 crosses per pair except of one pair (hybrid OFP × SN 2108) of plants where 11 crosses were made. Seeds from crosses were germinated and screened visually as previously described for GFP presence. Fertilility rates were determined by the number of attempted crosses divided by the total number of crosses attempted. Detectable gene flow was determined by dividing the total number of germinated GFP expressing seed by the total number of surviving seedlings.

**Table 1:** Greenhouse crosses performed with hybrid OFP plants

**Genotypes**^**a**^	**Plants crossed**^**b**^	**Total crosses**^**c**^	**Crosses forming seed**	**Total seed count**	**Germinated**	**Survived**	**GFP positive**	**Fertilization rate**	**Detectable gene flow**
(MS TN 90 × hybrid OFP)	8:8	96	2	51	0	N/A	N/A	2%	0%
(hybrid OFP × SN 2108)	8:8	95	18	34	14	12	10	18%	83%

^a^ Crosses listed by (female ♀ × male ♂).

^b^ Refers to numbers of each plant used in crosses with respect to the plant order in the genotypes column. 12 crosses were attempted between each pair of plants, except one pair of hybrid OFP and SN 2108 where 11 crosses were made.

^c^ A ‘cross’ is constituted by a pollen transfer from one plant to the flower of another plant.

### Statistical analysis

All analysis of variance (ANOVA) routines were performed using SAS (Version 9.3 SAS Institute Inc, Cary, NC, USA) using the MIXED procedure with a significance level of p < 0.05. When ANOVA results were found to be statistically significant, the least significant differences were used for mean separations.

## Results and discussion

Transformation of *N. tabacum* ’TN 90’ and *N. glauca* were successful except for *N. glauca* TD-GFP-H where multiple attempts failed to produce hygromycin-resistant plants. GFP was visible in leaves, stems, and roots (data not shown) and OFP was visible in pollen under a microscope (Figure 5) with the aforementioned filter set. GFP, regulated by the CaMV 35S promoter, was not visible in pollen in accordance with previous findings [19,20]. Highly fluorescent individual plants from the most fluorescent *N. glauca* TD-GFP-K lines were crossed with highly fluorescent TN 90 TD-GFP-H lines to ensure hybrids had both antibiotic resistance genes and would fluoresce brightly, thereby facilitating detection. Since we selected plants on the basis of green-fluorescence shoots, it is not surprising that pollen orange fluorescence was also bright in these lines. Hybrid OFP lines were 100% resistant to kanamycin and hygromycin when screened on MSO media containing both antibiotics (data not shown), indicating inclusion of both cassettes into the F_1_ hybrids.

Manual plant crosses revealed that the hybrids were able to backcross to a non-transgenic male sterile *N. tabacum* ‘MS TN 90’ (Table 1), forming entirely non-viable seed in 2% of the crosses (98% of the crosses produced no seed), thus restricting detectable transgene transmission rates in progeny to 0%. This result was in contrast to our previous findings where few viable seeds (2 out of 445 seeds from 96 crosses) were generated from a similar (MS TN 90 × hybrid) cross that employed a different construct using *mgfp5-er*[9]. In addition, we have determined that male fertility varies among hybrid lines from 0 to 3% [9]. When the fertile line, SN 2108, was used to pollinate hybrid OFP plants, limited seed set (19% of crosses) was observed. Only 10 germinated seedlings out of 12 expressed GFP, (83% detectable transgene transmission), indicating that transgenes might be segregating out of some hybrid OFP × SN 2108 progeny.

It was unknown if tdTomato-ER would be visible in the pollen of the *Nicotiana* hybrid as the plant largely produces immature pollen where many pollen mother cells cease to develop past the tetrad stage [21]. Many of the immature pollen grains apparently did not synthesize sufficient tdTomato-ER for visual detection. The FP was only obvious in larger, more mature hybrid OFP pollen and did not appear to fluoresce as brightly as TN 90 TD-GFP-K and *N. glauca* TD-GFP-H. The pollen-specific promoter *LAT52*, regulates gene expression during microspore mitosis, allowing transcription until anthesis [14,22]. Our observation of few mature fluorescent pollen grains produced in the hybrids demonstrates that the interspecific hybrid system could be a viable candidate for transgene bioconfinement.

## Conclusions

A bright orange fluorescent protein, tdTomato-ER, can be synthesized in pollen when its gene is under the control of the LAT52 pollen promoter. Fluorescently-tagged pollen is highly distinguishable from non-tagged pollen, and shows low autofluorescence. The plants produced in this study further increase the number of tools available for gene flow studies. Crossing studies demonstrated that hybrid OFP plants had low fertility and provided bioconfinement by limiting successful crosses made to the maternal line, *N. tabacum*. As pollen tracking is possible with this fluorescently tagged hybrid, more research is needed to determine the efficacy of pollen detection with this system and how it relates to bioconfinement in a field setting.

## Competing interests

The authors declare that they have no competing interests.

## Authors’ contributions

JHR: Performed all plant transformation experiments and transgenic line analysis, and drafted the document: REM: Conceived portions of the study, bred the original interspecific hybrid, produced the experimental hybrids, and performed manual greenhouse crosses. RJM: Coordinated the study and assisted with analysis. LLA created the vectors for transformation. ODC: Conceived portions of the study and assisted with coordination and execution of the study. HMD: Conceived portions of the study, coordinated the study, and provided critical review. CNS: Conceived portions of the study, coordinated the study, and assisted with revisions. All authors read and approved the final manuscript.
